# Thermosensitive Polymers and Thermo-Responsive Liposomal Drug Delivery Systems

**DOI:** 10.3390/polym14050925

**Published:** 2022-02-25

**Authors:** Waad H. Abuwatfa, Nahid S. Awad, William G. Pitt, Ghaleb A. Husseini

**Affiliations:** 1 Department of Chemical Engineering, College of Engineering, American University of Sharjah, Sharjah P.O. Box 26666, United Arab Emirates; g00062257@alumni.aus.edu (W.H.A.); nawad@aus.edu (N.S.A.); 2 Materials Science and Engineering Program, College of Arts and Sciences, American University of Sharjah, Sharjah P.O. Box 26666, United Arab Emirates; 3 Chemical Engineering Department, Brigham Young University, Provo, UT 84602, USA; pitt@byu.edu

**Keywords:** hyperthermia, thermosensitivity, liposomes, critical solution temperature, lysolipids, polymers

## Abstract

Temperature excursions within a biological milieu can be effectively used to induce drug release from thermosensitive drug-encapsulating nanoparticles. Oncological hyperthermia is of particular interest, as it is proven to synergistically act to arrest tumor growth when combined with optimally-designed smart drug delivery systems (DDSs). Thermoresponsive DDSs aid in making the drugs more bioavailable, enhance the therapeutic index and pharmacokinetic trends, and provide the spatial placement and temporal delivery of the drug into localized anatomical sites. This paper reviews the fundamentals of thermosensitive polymers, with a particular focus on thermoresponsive liposomal-based drug delivery systems.

## 1. Introduction

Nanoparticles-based drug delivery systems (DDSs) combat the adverse limitations associated with conventional treatment regimes, whether in cancer therapy, inflammatory conditions, or cardiovascular diseases such as hypertension and myocardial infarction [1]. Site-specific delivery of therapeutic dosages of the drugs to the diseased cells, while sparing the healthy tissues, is a key advantage of using DDSs. An effective DDS can retain, evade, target, and release its load with controllable and well-regulated kinetics [2].

Nanoparticles (NPs) incorporated in DDSs have been developed over the years, using a wide range of materials, and they exhibit unique chemical and physical properties, allowing them to deliver drugs with high precision. NPs can be broadly classified based on their chemical composition into organic and inorganic. Organic NPs can be lipid-based, such as liposomes and solid lipid NPs, or polymeric-based, such as micelles and dendrimers. On the other hand, inorganic NPs typically contain metals or metal-derivatives in their composition; these include quantum dots (QDs), gold NPs, carbon nanotubes (CNTs), metal organic frameworks (MOFs), and mesoporous silica. While some of these nanoparticles are in the development stage, others have progressed to preclinical and clinical trials. The efficacy of the different nanoparticles as DDSs varies, depending on their size, structure, and physical/chemical properties [3,4,5,6,7]. Such nanocarrier-based systems have especially shown notable implementation in the targeted treatment of cancer, by localizing the effect of antineoplastic agents to diseased tumor sites. Cancer cells induce nearby vascularization (angiogenesis) to meet the increased demands of oxygen and nutrient supply and to sustain rapid proliferation and survival pathways. The resulting intratumoral networks and neovasculature suffer from structural defects in the capillitial endothelium, as well as poor lymphatic drainage and aberrant fluid transport mechanisms [8]. The small size of the NPs allows them to benefit from the innate characteristics of tumors, and they can accumulate at the fenestrae of the tumoral matrices, in a phenomenon known as the enhanced permeability and retention (EPR) effect, which is the basis of passive targeting [9,10].

Besides the merits of passive targeting, active targeting techniques have emerged to further enhance the performance of such DDS. Uncontrolled cell proliferation stimulates the overexpression of certain biomarkers on the cells’ surfaces; nanocarriers can be decorated with motifs or moieties that specifically bind to these receptors. The specificity of active targeting emanates from the complementary arrangement of the overexpressed receptor and the targeting ligand, akin to the lock-and-key mechanism [11]. Besides the specific localization of the nanocarriers, surface functionalization can extend these carriers’ half-life and circulation time in biological systems. For example, PEGylation (modifying the surface with polyethylene glycol chains) has proven to be effective in shielding the nanocarriers from recognition and subsequent rapid elimination from the body by the immune system [12,13].

Conventional NPs generally release their loaded drugs passively, with no control over drug release. While some NPs show quick drug release and undesirable toxicity, the slow drug release from other NPs reduces drug efficiency. Controlling drug release from these NPs, after their accumulation at the targeted site, is essential to ensure spatial and temporal drug release at the diseased site. Advancements in nanomedicine have led to the development of smart NPs that can be triggered to release their load either endogenously (naturally within the body) or exogenously (remotely). Triggers such as ultrasound, light, pH, redox, enzymes, and heat have been widely investigated in the literature as promising stimuli modalities [14,15,16,17,18,19]. This review will focus on thermosensitive polymers and thermoresponsive NPs, particularly thermoresponsive liposomes, and the effect of temperature fluctuations on altering the structure of these NPs and the subsequent release of the loaded drugs.

## 2. Thermosensitive Polymers

Heat has been used as a therapeutic tool since the 1800s [20]. In 1866, the German surgeon Carl Busch reported the first account of effective long-term heating for damaging tumor cells without affecting healthy cells [20]. Subsequently, several early in vivo investigations were carried out to examine the thermosensitivity of tumors [21,22,23]. Raising the temperature of the cells to 40–43 °C, for around one hour, can destroy the structure of the cancer cells and affect other cellular processes, making the cells more prone to radiation and antineoplastic drugs [24]. Therefore, hypothermia is used as adjuvant therapy and is usually combined with other cancer treatments, such as radiation, chemotherapy, and immunotherapy [25]. However, it is extremely difficult to produce local hypothermia using the traditional methods without damaging the surrounding healthy tissues, due to prolonged exposure to elevated temperatures. Thermo-sensitive NPs are smart NPs designed to employ the local thermal energy to stimulate a spatial-temporal release of chemotherapeutic drugs at the desired site, while producing no adverse effects on the neighboring healthy tissues of normal temperature. Some NPs, such as liposomes and micro/nanogels, can be designed using a thermo-sensitive composition that changes in structure in response to the increase in the surrounding temperature, leading to a steady release of the encapsulated drug. For example, liposomes can be designed to ‘melt’ (change from the gel state to the liquid state) upon heating to a certain temperature. This type of liposomes is known as the traditional thermosensitive liposomes (TTSL). Thermo-sensitive micro/nanogels, on the other hand, are designed to change their volume by either shrinking or swelling when exposed to a specific temperature, known as the volume phase transition temperature or VPTT. Temperatures lower than the VPTT cause the polymer, forming the microgel, to swell up with the water. Temperatures higher than VPTT will result in causing the polymer to shrink [26,27] and release the encapsulated drug. Thermosensitive micro/nanogels response to the change in temperature is generally through reversible disruption of the hydrogen bonds located between the polymer and the surrounding water [28].

A major advancement in the field of thermoresponsive NPs was the development of different types of polymers that are sensitive to temperature (thermosensitive polymers). Incorporating those polymers within the structure of the NPs allows them to be sensitive or increase their sensitivity to a change in temperature, which alters their structure, resulting in them releasing their load in a controlled manner. NPs incorporating specifically tailored thermosensitive polymers can retain their payload at body temperature (37 °C) but deform and undertake a reversible volume phase transition upon local heating (~40–43 °C) [29].

**Figure 1:**
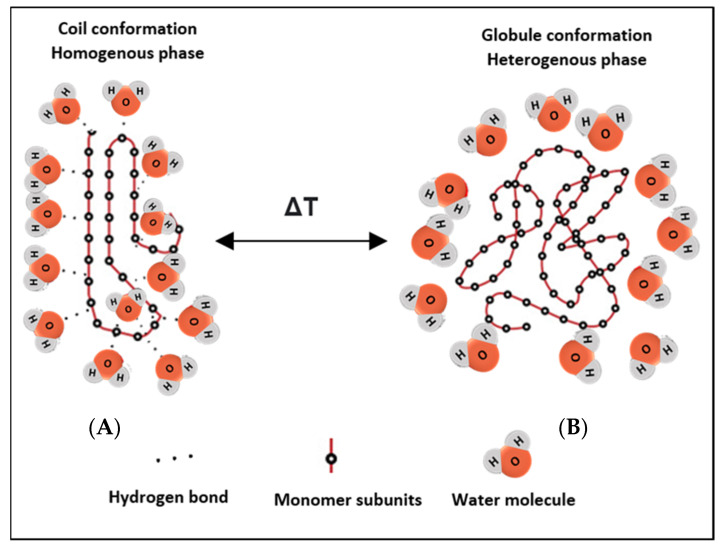
Schematic depicting the reversible phase transition from coil-to-globule and vice versa upon heating/cooling. (**A**) shows the hydrated state of the polymer, where hydrogen bonds are formed with surrounding water molecules at the hydrophilic ends, while (**B**) shows the nonhomogeneous state, where the chains dehydrate into globules and fold up, forming a water-rich and a polymer-rich phase. The change in conformation results from a change in the temperature of the system.

Potentiating drug release from nanocarrier-based delivery systems using temperature as a triggering modality is a well-established area of research and application. It is essential to fully comprehend the mechanism and rationale behind thermosensitive systems, to develop effective and sophisticated therapeutic platforms. Thermo-responsive nanocarriers incorporate thermosensitive polymers in their structures, which are designed to change their conformation upon exposure to heating/cooling. As the temperature of the surrounding solution increases, thermo-sensitive polymers show coil-to-globule transition. These thermo-responsive polymers usually contain hydrophobic and hydrophilic functionalities, to aid in designing their response. They can transition from the hydration to a dehydration state, in a ‘coil-to-globule’ shift from a homogenously dissolved state to a heterogeneously biphasic state, in response to a small change in temperature (Figure 1) [30]. Previous studies have also shown that both hydrogen bonding and hydrophobic interactions in polymer-solvent systems play a role in the transition from the hydrated random coil to the hydrophobic globule phase, as a result of temperature increase above the critical solution temperatures [31,32]. For example, thermosensitive polymers derived from neutral amphiphilic polymers such as acrylics, carry hydrophilic amide, ether, or alcohol groups and have hydrophobic hydrocarbon backbone chains [33]. Initially, when the homogenous monophasic system exists, the hydrophilic groups in the polymeric network interact readily with water molecules to form hydrogen bonds, resulting in randomly shaped hydrated coils. In response to heating/cooling effects, the conformation is altered where the hydrophilic units become isolated from the aqueous media, such that they are no longer accessible to the water molecules (forming local regions referred to as regions of hydrophobic hydration), initiating crystallization of the polymers to form a biphasic dehydrated nonhomogeneous system [33]. Disruptions to the shape and shrinkage in conformation result in drug release [34].

**Figure 2:**
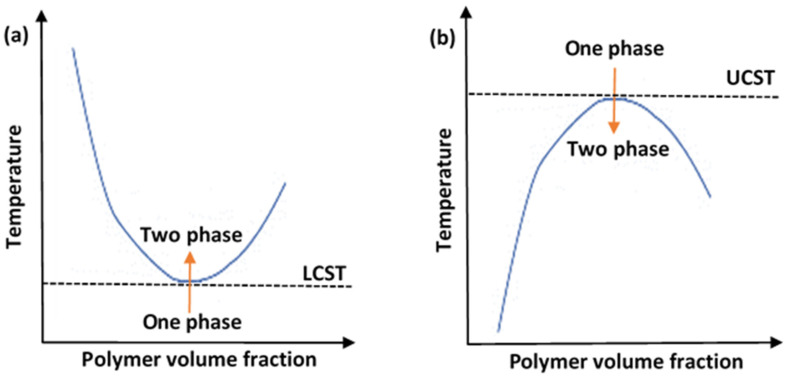
A diagram showing the phase transition behaviors of thermosensitive polymers in aqueous solutions, showing (**a**) lower critical solution temperature (LCST) system and (**b**) upper critical solution temperature (UCST) system.

An important characteristic of the solutions containing thermoresponsive polymers (polymeric solutions) is their critical solution temperature (CST). Generally, thermoresponsive polymers can be divided into upper critical solution temperature (UCST) and lower critical solution temperature (LCST) types. The phase diagram for each is depicted in Figure 2. Figure 2a shows a UCST system, whereby the polymers would be in the monophasic state above a certain temperate threshold. To alter the conformation of such systems, cooling would be necessary. These are referred to as “positive thermosensitive polymers”. On the other hand, Figure 2b shows an LCST system, and this is the preferred type employed in drug delivery applications. The LCST defines the limiting temperature, above which the system transitions to the binary phase and causes conformation contraction. These polymers are referred to as “negative thermosensitive polymers”. Thermodynamically, a UCST system is an enthalpy-driven system, where interpolymer interactions are more significant and dominant at low temperatures. In contrast, LCST systems are entropy-driven, where an increase in temperature causes the release of the hydrated water molecules (hydrophobic interactions dominate) [34].

**Figure 3:**
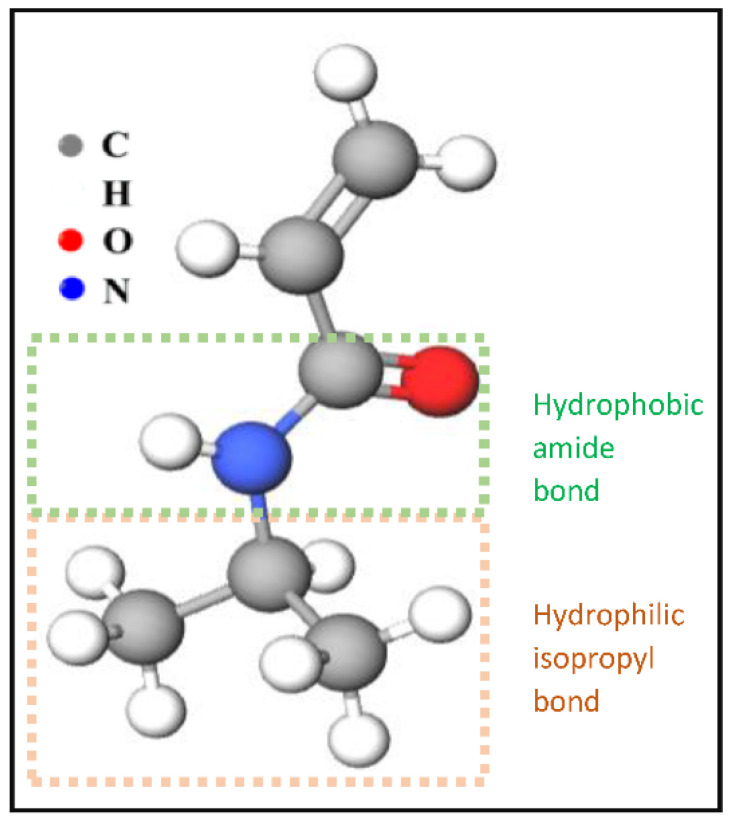
The chemical structure of NIPAAm monomers.

**Figure 4:**
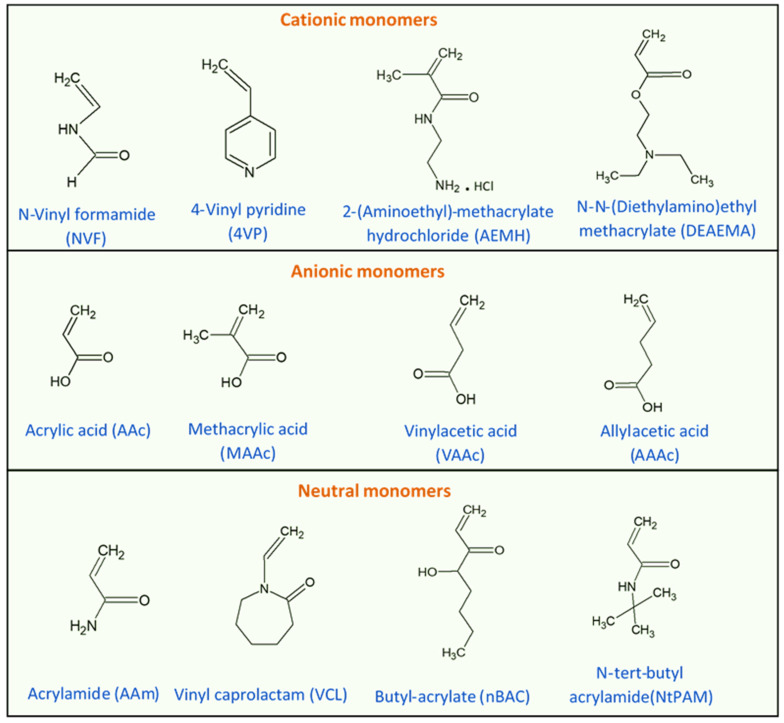
Chemical structures of monomers commonly used in the synthesis of PNIPAAm copolymers with modulated thermoresponsive properties, classified based on their charge.

One important LCST polymer is a polyalkylacrylamide derivative, namely poly(N-isopropyl acrylamide) (PNIPAAm). This polymer forms soluble chains in the water below its LCST due to hydrogen bonding between the water and the polymer’s polar groups. Above 32 °C, the waters molecules are expelled from the network, causing the structures to contract, by dehydration of the isopropyl groups [29,30]. The chemical structure of the N-isopropyl acrylamide (NIPAAm) monomer is shown in Figure 3. Generally, the volume phase transition temperature (VPTT) and behavior of thermosensitive hydrogels/microgels can be tailored by changing the balance of the hydrophilic and hydrophobic groups or by introducing an electrostatic charge into the polymer that would influence the polymer–polymer and water–polymer interactions [35]. Thus, copolymerization of PNIPAAm with different monomers that induce different conformational and swelling/de-swelling behaviors from the pure homopolymers conformations is regarded as a flexible strategy to modulate the thermoresponsivity of PNIPAAm-based copolymer systems [35]. The copolymerization monomers can be positively, negatively, or neutrally charged, where philicities, polarities, and concentration come into play. These initiators can be added to the precursor mix during synthesis [35]. Figure 4 shows some of the most common comonomers used to synthesize PNIPAAm copolymers, classified based on charge. For example, the VPTT of PNIPAAm can be changed to ~45 °C by copolymerizing with hydrophilic co-monomers such as acrylamide (AAm), which increase the copolymer chain stiffness and hydrophilicity, as well as limit intramolecular interactions [36]; or with acrylic acid (AAc), which due to its carboxylate groups provides additional repulsive electrostatic interactions that result in a two-step temperature-induced conformational change [37,38]. Polymer size was found to increase with the increase in AAc concentration, as well as a shift in VPTT to a higher temperature due to the hydrophilic nature of AAc [39].

Although PNIPAAm-based copolymers have garnered great interest in research, there are other thermosensitive particles derived from other types of polymers. For example, Pluronic F127 (Poloxamer 407) is an amphiphilic ABA-type triblock copolymer composed of poly(ethylene oxide)_98_–poly(propylene oxide)_67_–poly(ethylene oxide)_98_ blocks (PEO_98_–PPO_67_–PEO_98_) [40]. It is an attractive biomaterial for the synthesis of thermosensitive drug delivery systems, as it was approved by the FDA for human use. In addition to their reversible gelation capabilities, non-toxicity, biodegradability, and biocompatibility, Pluronic F127-based systems exhibit prolonged drug residence times [40,41]. Table 1 presents some studies that proposed Pluronic F-127-based systems for different drug delivery applications. The main mechanism driving the volume transition above the LCST is attributed to thermal-induced collapse, due to micellization and self-association of the crosslinked Pluronic copolymers dominated by inward hydrophobic interactions [42,43,44]. Such systems with flexible thermal response windows and physical properties have promising potentials for diagnostic and therapeutic applications.

Thermosensitive nanoparticles (TNPs) benefit from their small size (100–200 nm) in penetrating biological barriers, aiding in the localized delivery of agents and drugs. In addition, the small size of the TNPs allows for rapid reaction to physical changes, as the relaxation time of the volumetric change is directly proportional to the particles’ radius squared (at the critical point) [46]. A rapid transition rate at the critical solution temperature (CST) is always favorable in practical applications. Moreover, TNPs exhibit high specific surface areas compared to particle size, providing a larger number of active sorption sites, which aids in their uptake and biological mobility. Another important property is dispersity of the size distribution, as monodisperse populations exhibit better reaction kinetics to changes at the CST. The current synthesis routes are mostly successful in producing populations with small polydispersity indices, which are calculated as the ratio of weight-average diameter to the number-average diameter of the particles in the distribution [47].

To form thermosensitive polymeric particles with desirable properties, synthesis can be initiated from monomers, polymeric solutions, or macrogels. The most common synthesis routes start with vinyl monomers, which can be neutral or charged. Moreover, a polymeric solution, which contains a crosslinking agent, can be a synthesis precursor or a macrogel that can be physically reduced to form microgel particles [35]. Pelton and Hoare [48] broadly classified the synthesis routes of thermosensitive polymers into three approaches, which are homogenous nucleation, emulsification, and complexation. In the homogenous nucleation approach, the synthesis precursor is a homogenous polymeric solution that contains at least one type of monomer and one crosslinker substance. Including more monomer types increases the complexity of the final product and allows for facile functionalization [35]. The process can be carried out through emulsion polymerization or surfactant free emulsion polymerization (also known as precipitation polymerization). In the emulsion polymerization route, the starting solution contains a suspension of large monomer droplets that are stabilized by surfactant molecules. An example is the preparation of colloidal dispersions of PNIPAAm [49], starting with a precursor solution containing two types of monomers: NIPAAm and methylene-bis-acrylamide, and sodium dodecyl sulfate (SDS) surfactant. The transition temperature of the produced particles was 32 °C, below which the polymer chains would be swollen. Above it, they would shrink due to water rejection, decreasing the average diameter by 2-fold, as a function of the experimental parameters tested. The thermoresponsive behavior and physical properties of the particles was found to be dependent on the concentration of SDS during the polymerization process. Moreover, the particles exhibited a charge due to the carboxyl and sulfate groups from the initiator, which had a noticeable impact on the swelling behavior of the particle at low electrolytic ionic strengths only. This method is widely used because it is robust, versatile, and well-understood. An example of surfactant-free synthesis is the preparation of latex dispersions using monomers of NIPAAm, acrylamide, and N,N′-methylenebisacrylamide [50]. Potassium persulfate was used as an initiator for the free radical polymerization reaction. The precursor solution also contained certain amounts of N,N′-methylenebisacrylamide as a crosslinker. The resulting hydrogels decreased by 10-folds in average diameter upon heating above the LCST. The LCST was found to be a function of acrylamide concentration.

Besides the homogenous nucleation approach, emulsification and complexation are other common routes for thermosensitive particle synthesis. Emulsification is also referred to as inverse emulsion polymerization or mini-emulsion polymerization. A dispersion of hydrophilic monomers in an aqueous phase would be emulsified and polymerized in a continuous non-aqueous phase [35]. Typically, a pre-gel solution (either free monomers or a polymeric solution) is emulsified in oil or any other non-aqueous medium. The droplets are polymerized and crosslinked to form the thermosensitive particles. Polyacrylic acid-based microgels were synthesized using this method by using cyclohexane as the nonpolar continuous medium [51]. Chen et al. [52] characterized the emulsification process of acrylic acid monomers into micelles and concluded that the reaction kinetics (i.e., rate of polymerization) were a direct function of the starting concentration of the monomer. In addition, the monodispersity of the size distribution was dependent on the concentration of the crosslinking agent. The copolymers produced via this synthesis route were robust and less susceptible to coagulation, as the crosslinking agent copolymerizing interfacially yielded hard particles. Meanwhile, complexation depends on forming a colloidal polyelectrolyte complex comprised of two dilute hydrophilic polymers, where one is in excess abundance compared to the other, in order to provide electrostatic stabilization. Feng and colleagues [53] studied the complexation interactions between poly(vinyl amine) and carboxymethyl cellulose and concluded that the mean size of the synthesized complexes was insensitive to the mixing ratio of each polymer, although the particle size distribution was broad. Moreover, mixing the two polymers at different ratios resulted in soluble complexes, colloidal complexes, and macroscopic precipitates; although, stable colloidal complexes were formed only when one of the polymers was provided in excess of the other, in order to contribute to the electrosteric stabilization of the mix. However, the two drawbacks of this approach are (i) the difficulty in separating the excess polymer, and (ii) the high polydispersity index in the particle distribution.

## 3. Thermoresponsive Liposomal-Based Drug Delivery Systems

Liposomes are nanosized carriers that resemble in their structure those of cell membranes. They have a lipid bilayer architecture comprised of phospholipids that assemble into concentric spherical shells of lipid bilayers with each bilayer’s inner and outer surfaces made up of hydrophilic heads and the hydrophobic tails arranged inside the bilayer and, hence, shielded from the surrounding aqueous environment [6]. This distinguishing arrangement allows for the encapsulation of hydrophobic and hydrophilic drugs simultaneously, expanding the potential prospects of exploiting liposomes in the field of chemotherapeutics delivery. Moreover, the encapsulated drugs are protected by the liposomes’ physiological stability and biocompatibility; and are, hence, less susceptible to degradation and dilution upon administration [54,55,56].

### 3.1. Traditional Thermosensitive Liposomes (TTSL)

Given the merits of these nanocarriers, different modifications have been proposed and assessed to further exploit these advantages and improve their therapeutic performance. Temperature-induced drug release from liposomes is a concept that emerged in the late 1970s. The first thermosensitive liposomes (TSLs) were introduced in 1978 by Yatvin and colleagues [57], who were able to release neomycin from liposomal carriers at different temperatures to hinder in vitro protein synthesis pathways. These liposomes are designed to ‘melt’ upon heating above a certain temperature threshold, called the transition temperature (Tc). They undergo a reversible thermotropic ‘gel-to-liquid crystalline’ transition, with gel being the ordered state and liquid crystalline being the disordered state [57]. The thermosensitive formulation, which consisted of 1,2-Dipalmitoyl-sn-glycero-3-phosphocholine (DPPC) and 1,2-Distearoyl-sn-glycero-3-phosphocholine (DSPC) (molar ratio 3:1) showed 100-times more drug release at 44 °C than at 37 °C. In the gel phase, the phospholipids are aligned and well-packed together in a perpendicular orientation with respect to the surface plane of the lipid bilayer, resulting in a minimal cross-sectional area, favoring thermodynamic stability. As such, the highly-ordered structure inhibits inter and intramolecular dynamics, creating an impermeable barrier that completely separates the intra- and extravesicular domains. As the temperature of the system approaches Tc, the single bonds between the carbons in the hydrocarbon chains change the configuration from trans to gauche, and the lipid heads become more mobile. Domains of highly disordered and random incompatible packing start to exist at the interfaces between still-solid lipids and ones that have melted, and these locations become permeable. Those microscopic regions are named ‘grain boundaries’. Grain boundaries separate the domains containing phospholipids in the gel phase from the other domains containing phospholipids in the liquid phase located within the same liposomal membrane. As the temperature exceeds the Tc, the membrane becomes fully fluidized and leaky, to release the cargo [57,58].

While DOX release from the beforementioned liposomal system was successfully induced at temperatures below 42 °C, in vitro studies showed that continuous heating at temperatures exceeding 42 °C is needed to release at least 50% of the drug load [59,60]. Such conditions are unfavorable in clinical settings, as higher thermal doses are associated with thermal necrosis complications, besides the practical difficulty of raising the tumor tissue temperature to such levels without pain to the patients or surface burns. Other traditional thermosensitive liposomal systems (TTLSs) are listed in Table 2. Nonetheless, most of these liposomal formulations have been abandoned due to several significant limitations that hinder their progress in research and application. The impractical transition temperatures at which TTSLs respond cannot be achieved in clinical settings, impeding their use [58]. Moreover, the lack of burst release kinetics (slow-release >30 min), formation of aggregates, and thermodynamic instability contributed to the devising of optimized formulations that can respond to hyperthermia at milder conditions (39–42 °C). Thermoresponsive systems within this therapeutic window exploit the advantages of hyperthermia (i.e., sensitize tumor cells to antineoplastic agents), while precluding its adverse side effects (i.e., thermal skin damage) [60]. The two main approaches to achieving the desired optimized outcomes of thermosensitive liposomal systems (TLSs) are the introduction of lysolipids into the formulations, and modification of the bilayer by the inclusion of membrane-disruptive polymers [60].

### 3.2. Lysolipid Thermosensitive Liposomes (LTSL)

The chemical composition of TTSLs can be favorably altered by incorporating thermosensitive phospholipids, lysolipids, which contain one acyl chain. These lipids are non-cylindrical (larger head size than the single tail). Thus, they can be easily incorporated into the liposomal membranes, changing such characteristics as morphology, stability, permeability, and Tc [69]. Generally, the transition temperature of phospholipids is a function of the hydrocarbon chains length, electrostatic properties of the head groups, and the acyl chain saturation levels. The membrane curvature changes according to the formulation chemistry, particularly the concentration of lysolipids in the formulation. The lysolipids exhibit positive intrinsic curvatures that tend to form stabilized defects in the bilayer. The thermally-activated drug release mechanism is based on the hypothesis that the inclusion of lysolipids introduces pores, fenestrations, and grain boundaries (microscopic domains of disorder) into the membrane structure upon phase transition; thus, creating higher permeability toward the enclosed drugs. The burst or rapid release kinetics provided by lysolipids are essential to the pertinence of the proposed nano-delivery platform [69].

**Figure 5:**
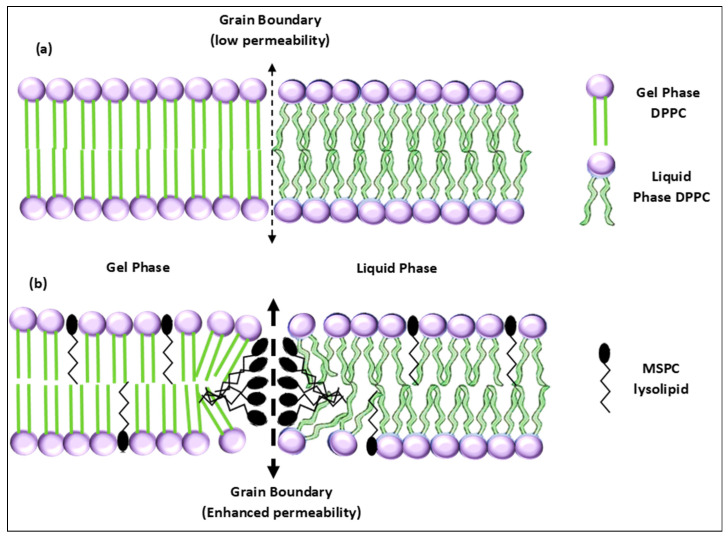
A schematic showing phase transition of the lipid bilayer in (**a**) traditional TSLs and (**b**) LTSLs. Inclusion of the lysolipid MSPC increases the membrane permeability due to introducing planar vacancy defects.

Several studies [69,70,71] examined the DOX fraction release as a function of varying lysolipid concentrations in formulations, such as Mills and Needham’s [70] studies on liposomes containing 3.8 mol% DSPE-PEG2000 with varying mono-stearoyl phosphatidylcholine (MSPC) content up to 15 mol%. Almost 80% release from all formulations was observed within the first 20 s of heating to 41.3 °C. Blood flow pattern analyses and tumor hemodynamics studies, e.g., Chen et al. [71] and Dewhirst et al. [72], confirmed that the red blood cell (RBC) velocity within tumoral microvessels exceeds 20 s (~0.5 mm/s, considering the volume of the lump); thus, thermally triggered DOX release within 20 s is sufficient for the drug to accumulate and be biodistributed at the tumor site. Such liposomes are commonly referred to as ‘lysolipid thermosensitive liposomes’ (LTSL) (Figure 5). The idea of this compositional modification was introduced back in the late 1990s, by Needham and his colleagues [2,69,70].

Needham et al. [73] synthesized LTSLs for controllable DOX release (ThermoDox^®^) at mild hyperthermic temperatures (39–40 °C). They tested their in vitro and in vivo efficacy and performance in thymic nude mice bearing FaDu human squamous cell carcinoma xenografts. The researchers incorporated 10 mol% monopalmitoyl phosphatidylcholine (MPPC) into the liposomal chemistry to induce an amplified increase in membrane permeability during the phase transition; thus, the release onset started at a slightly lower temperature (~39 °C). Three stealth formulations were synthesized and tested, non-thermosensitive liposomes (NTSLs) (HSPC:cholesterol: DSPE-PEG-2000 75:50:3), TTLSs (DPPC:HSPC:cholesterol:DSPE-PEG-2000 100:50:30:6), and lysolipidified thermosensitive liposomes (LTSLs) (DPPC:MPPC: DSPE-PEG-2000 90:10:4). The burst release from TTSLs occurred between 41 to 43 °C, followed by a slow release that extended over ~30 min, whereas NTSLs showed insignificant release dynamics within the physiological temperature range (37 °C). The results were promising for the novel therapeutic platform, and the enhanced release kinetics (burst drug release within seconds) of the LTSLs were attributed to (1) the increased bilayer permeability induced by the incorporation of MPPC, which introduced defects into the membrane; and (2) the different dissociation and shearing of the lipids as the first molten layer was able to desorb from the bilayer conformation. In terms of thermal dose, the equivalent minutes remarkably decreased from 7.5 to 0.08 min for treatment with TTSLs and LTSLs, respectively. The reduced thermal dose value falls well below the limit for the onset of thermal necrosis. Treatment with the LTSLs resulted in complete regression and tumor arrest in the mice for up to 2 months of observation, evidencing the therapeutic potential of LTSLs [73].

A subsequent study by Kong et al. [74] examined the changes in hyperthermia-induced cytotoxicity, drug interactions, liposomal accumulation, and release kinetics of different formulations on athymic nude mice bearing FaDu human tumor xenografts. The different groups were treated with a cumulative DOX dose of 5 mg/kg by a single i.v. administration. To understand the effects of hyperthermia, some groups were treated at 34 °C and others at 42 °C. The local drug biodistribution assessment in the tumors showed the highest DOX accumulation of about 25.6 ng/mg using the LTSLs with hyperthermia. The groups exposed to temperature stimulus exhibited higher DOX accumulation in the tumors compared to the non-heated groups. Groups treated at 42 °C showed significant tumor volume reduction compared to the non-heated groups. Similarly, DOX fluorescence analysis of tumor sections treated with TTSL and LTSL at 34 °C and 42 °C supported the conclusion that combining LTSLs with hyperthermic treatment showed superior tumor growth inhibition activity in comparison to the other treatment combinations [74].

Li et al. [75] considered the effects of combining a two-step clinically used mild hyperthermia treatment (HT) with liposomal chemotherapy on enhancing drug accumulation and bioavailability at the tumor site. DOX-loaded thermosensitive liposomes were used, where the first HT treatment (~41 °C) was intended to enhance the accumulation at the tumor matrix, while the second (~42 °C) aimed at stimulating drug release from the nanocarriers. The liposomes were made thermoresponsive to mild hyperthermia by manipulating the DSPE content in the formulation, which increased the transition temperature, due to the structural defects it introduced at higher concentrations. The results illustrated the time-dependency of the release kinetic from the liposomes at two different temperatures (37 and 42 °C) and showed the efficacy of the novel approach in arresting metastasis and inhibiting tumor growth in nu/nu mice bearing Human BLM melanoma cells. The two-step HT model effectively increased drug bioavailability and enhanced the controlled release kinetics, offering a promising approach within attainable clinical conditions.

**Figure 6:**
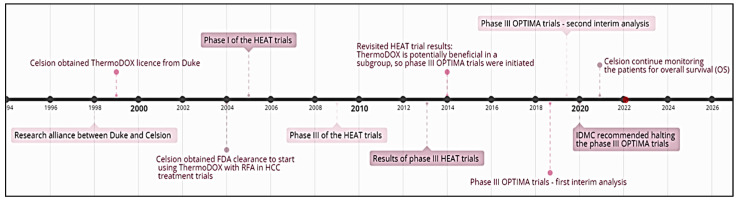
Timeline detailing the progress of ThermoDOX in clinical trials from 1998 onwards.

In 2005, and following initial preclinical analyses, Celsion Corporation began clinical experiments to prove the effectiveness of ThermoDOX in inhibiting tumor growth, under mild hyperthermia conditions, and to obtain regulatory approval. ThermoDox uses lysolipid thermally sensitive liposomes’ technology to encapsulate DOX. This heat-responsive liposome is designed to rapidly change structure in response to a rise in temperature to 40–45 °C. Upon heating, pores are created on the phospholipid wall surrounding the liposomes, releasing DOX into the targeted site [76]. ThermoDOX is the only thermoresponsive liposomal formulation that has made it to clinical trials (Figure 6), but not yet to commercial markets. This formulation has received orphan drug designation from the European Commission (EC), USA orphan drug status, and FDA fast track designation, for hepatocellular cancer treatment [77]. The aim of the first clinical trial was to evaluate ThermoDOX safety and clinical feasibility in conjunction with radiofrequency ablation (RFA) to create targeted thermal zones for the treatment of hepatocellular carcinoma (HCC). Combining ThermoDox with RFA presented a multi-modal treatment, where RFA is used to destroy the cancer cells, while also heating the vasculature surrounding the tumors and, thus, triggering DOX release from the thermosensitive liposomes circulating inside the heated vasculature. This first clinical trial was called the ‘HEAT’ trial and included 24 patients. Due to the promising early results and the urgent need for an effective treatment for HCC, the trial was directly fast-tracked to phase III in 2009. However, the HEAT trials did not meet the endpoints for progression-free survival (PFS). It was suggested that the failure of the HEAT trials in meeting their outcomes was due to clinical trial design limitations such as poor drug choice, inadequate treatment schedules, unoptimized heating protocol, and an inappropriate selection of the primary endpoint (progression-free survival (PFS) rather than overall survival (OS)). The major trial results are summarized in Table 3.

Analyzing the results obtained during the HEAT trial revealed that patients who received a prolonged exposure to the RFA waves (minimum dwell time of 45 min) have benefited from this therapeutic modality. Those promising results lead to launching a new Phase III clinical trial (OPTIMA trial) (NCT02112656) in 2014, which employed a standardized heating protocol with a minimum RFA dwell time of 45 min. However, in February 2021, Celsion Corporation terminated this trial, as it failed to demonstrate that combining ThermoDox with RFA provided measurable survival benefit over cancer treatment using RFA alone.

Currently, there are ongoing phase I trials recruiting candidates, where ThermoDOX is combined with other heating modalities. Trial (NCT04852367) proposed using focused ultrasound (FUS) to create the thermal zones for the treatment of non-resectable pancreatic cancer (PanDOX) [78], while trial (NCT03749850) is a feasibility study that proposed using magnetic resonance-guided high intensity focused ultrasound (MR-HIFUS) with cyclophosphamide administration, alongside thermosensitive liposomal DOX treatment [79].

**Table 3:** Summary of ThermoDOX clinical trials.

Study/ClinicalTrials ID	Status/Phase	Intervention	Indication	Remarks	Ref.
HEAT/NCT02181075	Completed/phase III	Lyso-thermosensitive liposomal doxorubicin (ThermoDOX) in conjunction with radiofrequency ablation (RFA)	Non-resectable hepatocellular carcinoma (HCC)	A total of 701 patients were divided into two experimental groups: 354 patients received a single ThermoDOX intravenous infusion (50 mg/m^2^) 15 min prior to RFA, while 347 were given a sham infusion of 5% Dextrose (placebo) 15 min before RFA. RFA was used to induce a thermal zone at the tumor site, where the entrapped doxorubicin was subsequently released from the liposome. Although the combination of ThermoDOX with RFA was safe, it did not increase the progression-free survival (PFS) and overall survival (OS) in the overall study subjects.	[80]
OPTIMA/NCT02112656	Completed-phase III	ThermoDOX followed by standardized RFA	Non-resectable HCC	A total of 554 subjects enrolled in the trial; divided into an experimental group that received 50 mg/m^2^ doxorubicin, while the control group received a dummy infusion. RFA was initiated at least 15 min after drug administration and completed within a maximum of 3 h from administration time. RFA exposure was for a minimum of 45 min. CT scanning and MRI imaging were used to gauge the effectiveness of RFA. The second interim data analysis was unexpected, due to the consecutive death of 26 cases. The trial marginally crossed the futility preset boundary value of 0.900 by 0.003, which led to recommendations from the Independent Data Monitoring Committee (IDMC) to terminate the trial in 2020. However, the Celsion Corporation company announced that they will continue monitoring the patients for overall survival (OS).	[81,82]
TARDOX/NCT00617981	Completed/phase I	ThermoDOX followed by focused ultrasound (FUS) exposure	Unresectable and non-ablatable primary or secondary liver tumors	The study was conducted in two parts, run in parallel: part I had 6 patients, while part II had 4. Optimized FUS parameters from part I were used in part II, determined based on real-time thermometry. Patients received ThermoDox^®^ intravenously, at a dose of 50 mg/m^2^, followed by FUS exposure. Reported tumor biopsy results showed a 3.7-fold increase in intratumoral doxorubicin accumulation in patients treated with FUS, proving this combination treatment as safe, effective, and feasible for further clinical investigation. While no treatment-related deaths occurred, severe adverse events were registered in some patients (e.g., transient neutropenia, anemia).	[83]

### 3.3. Polymer Thermosensitive Liposomes (PTSL)

**Figure 7:**
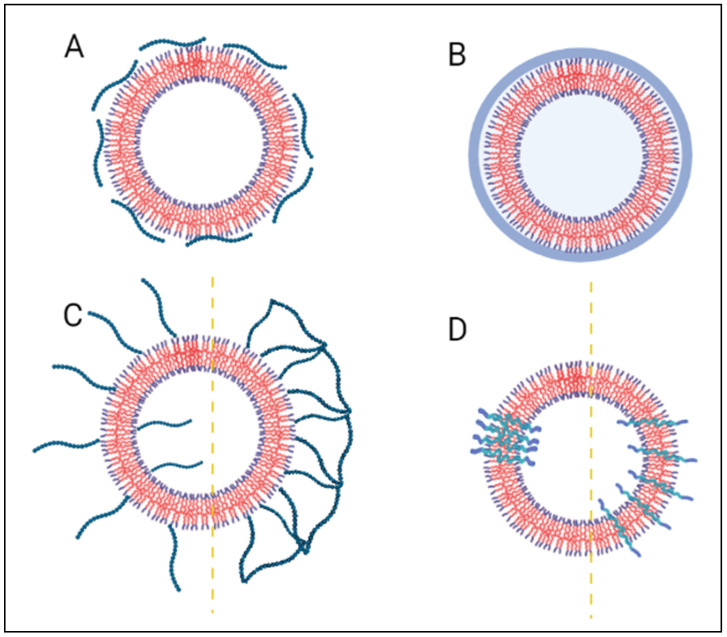
Hydrophilic thermosensitive polymers can (**A**) adsorb on the liposome surface, (**B**) encapsulate the liposome, (**C**, left) be covalently bonded to the polar phospholipid heads, or (**C**, right) reticulate to form fused networks on the liposome surface. Amphiphilic thermosensitive liposomes either (**D**, left) segregate in distinct domains, or (**D**, right) uniformly distribute in the lipid bilayer.

Another approach to sensitizing liposomes to lower mild hyperthermia conditions involves integrating thermosensitive LCST polymers into the liposomal structures. The introduction of polymers also addresses concerns about eventual in vivo and in vitro thermosensitivity-loss of LTSLs, as the lysolipids tend to desorb and leach out from the liposome bilayer, leaving behind fenestrae open to the surrounding biological milieu [84]. Premature drug release from LTSL was demonstrated in vivo, as about 50% of the encapsulated DOX was released within 1 h of administration in mice kept at 36.5–37.5 °C [61]. In comparison, up to 80% was released in vitro within half an hour, when tested in serum at physiological conditions [85,86]. Thus, conjugating or polymerizing the liposomes with thermosensitive polymers is a promising approach that overcomes the drawbacks of older designs. These synthetic polymers can be used to introduce thermosensitivity to the non-thermosensitive formulation or augment the thermo-responsiveness of already thermosensitive formulations. At temperatures below the LCST, the polymers are completely hydrated, hindering interactions with the extra-liposomal environment and preventing cargo release. As the liposomes experience an increase in temperature, the polymers shrink and condense into their dehydrated globular forms, disrupting the membranes’ stability and releasing the drug load [87]. These polymers can be easily tuned to respond to the desirable range of temperatures, thereby impacting the liposomal responsivity as well. Such liposomes are commonly referred to as ‘polymer thermosensitive liposomes’ (PTSLs). As previously discussed, various thermosensitive polymers exist in research and can be modified according to the requirements. Liposomes surface modification with thermosensitive polymers dates to 1991, where Ringsdorf and colleagues [88] tried inducing reversible conformational transitions in liposomal membranes by incorporating hydrophobic PNIPAAm chains onto them. This study was fundamental in outlining the basis of coil-to-globule chemistry in the science of polymers. Figure 7 illustrates the different ways polymers can be incorporated into liposomes. Hydrophilic thermosensitive polymers can be physically adsorbed on the liposome surface (Figure 7A), polymerized to entrap the liposome inside (Figure 7B), covalently bonded to the phospholipid heads (Figure 7C left), or polymerized into fused networks on the surface of the liposome (Figure 7C right). Furthermore, amphiphilic thermosensitive polymers can either be separated in segregated domains (Figure 7D left) or homogenously distributed through the liposomal bilayer (Figure 7D right) [89].

Research in this area has blossomed due to the merits of this approach, which include facile synthesis schemes, flexibility in tuning the properties, and highly efficient systems, which can cater to the burst release requirements. Kim et al. [90] reported that copolymerization of NIPAAm and AAc, then mixing the result into liposomes primarily composed of egg phosphatidylcholine (PC) and DPPC, resulted in a highly-controlled thermoresponsive system. Similarly, Han and colleagues [91] successfully modified DPPC, HSPC, and cholesterol (56:28:17 mol%) based liposomes with PNIPAAm-AAc mixed at a ratio of 83 to 17 (mol/mol%). The PTSLs showed remarkable release of encapsulated DOX, corresponding to almost 65% of the load after 5 min of hyperthermal exposure at 39 °C. At temperatures less than that, i.e., 37–38 °C, the carriers were able to retain almost 90% of their contents. To summarize, functionalizing liposomes with thermosensitive polymers can yield highly controllable therapeutic platforms with desirable tunable properties. Table 4 presents some studies which investigated the effects of comonomers choice for affecting PNIPAAm polymer thermo-responsiveness, and which extend to the liposome’s thermosensitive functionality.

## 4. Heat-Triggered Release Modalities

**Figure 8:**
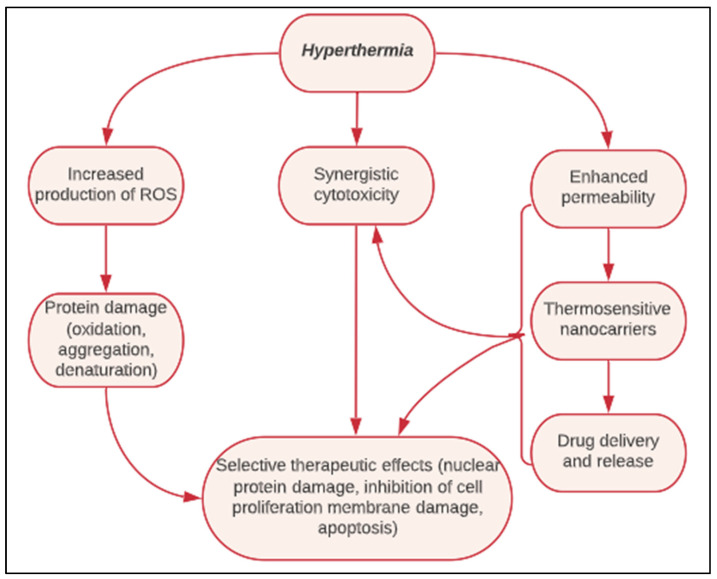
Schematic illustrating the multifactorial effects of temperature-triggered hyperthermia, from its stand-alone cytotoxicity to inducing synergistic cytotoxic effects when combined with drug delivery systems.

Upon accumulation of the thermosensitive nanocarriers at the diseased site, they need to be activated to release their contents in a spatially and temporally controlled manner. The process necessitates differentially elevating the temperature of the targeted region, while maintaining the surrounding tissues at normal physiological conditions. The DDS needs to be thermally responsive within the allowable therapeutic window; defined as between 40 to 43 °C for clinical mild hyperthermia applications. Heating the tumor to a higher temperature will produce cell necrosis, and the cells will disintegrate in response to the high temperature, rather than the treatment itself [95]. When administered with suitable parameters, temperature stimuli can cause hyperthermia (Figure 8), which can aid in (i) increasing blood flow and perfusion, (ii) augmenting the enhanced permeability and retention (EPR) effects of leaky vasculature by increasing interstitial fluid flow and microconvection transport dynamics, (iii) inducing gaps and pores in the endothelial lining of the targeted area, (iv) sensitizing cells to cytotoxic agents, and (v) in chemotherapy applications, the intratumoral matrix can become hypoxic, more acidic, and deprived of the necessary components for survival pathways [96]. On the cellular level, nuclear protein damage and alterations to cellular homeostatic pathways are considered direct effects of hyperthermia, which lead to inhibition of DNA replication and repair and eventual apoptosis [97]. Many studies have linked heat-induced cytotoxicity with the increased production of reactive oxygen species (ROS) such as hydrogen peroxide, which can potentially cause oxidative damage to the cells’ proteins, lipids, and nucleic contents, as well as disturbing mitochondrial potential and activity [69,98].

**Figure 9:**
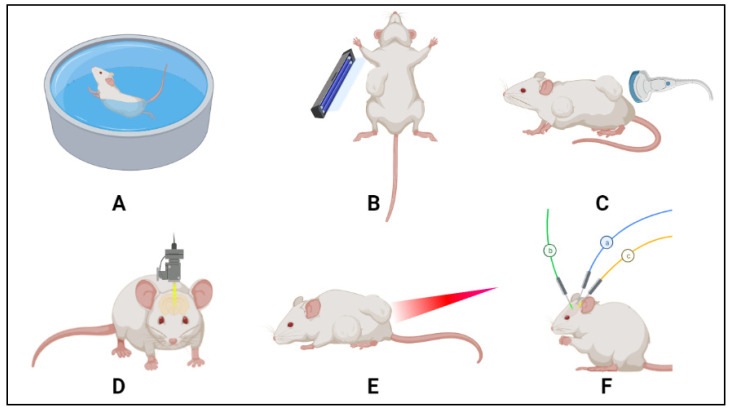
Different heating modalities for triggering release from TSLs: (**A**) water bath, (**B**) cold lamp, (**C**) US, (**D**) laser, (**E**) microwave radiation, and (**F**) interstitial methods (e.g., radiofrequency).

Hyperthermia causes vasodilation in normal tissues, which increases blood flow, depending on duration, intensity, and heating mode. In tumors, the trends are more complicated, given the heterogeneity within the tumor matrix itself, as well as the different pathological characteristics of the various cancers. An early study by Song [96] showed that the temperature in tumors rises upon heating more than in normal tissues, because tumors have a defective vasculature that does not experience a significant increase in blood flow (to aid in heat dissipation); hence, the heat accumulates more at the tumor site. The vascular damage, suboptimal conditions, and changes in oxygenation of the tumor tissues upon hyperthermia treatment affect the drug biodistribution and effectiveness; thus, it is important to consider the exposure dose behavior of the designed thermoresponsive systems, especially for biomedical applications such as drug delivery or imaging applications. Different heat modalities are used for triggering drug release from TSLs (Figure 9).

Regional hyperthermia induced by superficial heating of the targeted areas using a water bath has been extensively used in in vivo studies [99,100,101,102,103]. In this setup, small animals are usually fixed on specially designed holders to expose certain areas of the body to the heated water, while minimizing its contact with the surrounding skin. The main drawbacks of using water bath heating are (i) the limited penetration of the heat, such that it can only effectively heat superficial tumors, but not deep-seated ones; and (ii) poor localization of heating, as surrounding tissues eventually experience elevations in temperature upon extended exposure [104]. Superficial heating can also be achieved using external applicators, such as cold lamps that emit visible light (350–700 nm) [64,104], near-infrared (NIR) lasers (~800–1000 nm) that can reach ~0.5 cm deep tumors [105], or FDA-approved microwave devices (30 to 0.03 cm) [106].

To reach deeper tumors, localized interstitial hyperthermia can be achieved by using minimally invasive antennas or applicators, which use different sources for heating, such as ultrasound or radiofrequency radiations. To ensure homogenous heating throughout the matrix of the tumor, electrodes with expandable extensions or several applicators must be inserted simultaneously under imaging guidance. This method is limited to small tumors (<5 cm in diameter) that are seated in accessible locations for insertion of the appendages (e.g., prostate, breast, neck) [60]. Several studies [107,108,109,110] have demonstrated the use of high intensity focused ultrasound (HIFUS) to effectively trigger release from TSLs and showed that this approach is advantageous due to its high tunability, temporal control, and well-understood kinetics. Tumor tissues can absorb the acoustic energy and convert it to thermal energy, resulting in heat accumulation inside the targeted mass, which can eventually disturb the thermoresponsive nanocarriers. A major drawback of HIFUS applications is the lack of a comprehensive HIFUS system that uses noninvasive temperature monitoring methods (rather than invasive thermocouples) for more patient-friendly applications [60]. To address this issue, magnetic resonance-guided focused ultrasound (MRgFUS) has emerged as a solution that allows for image-guided thermal treatment and exploits MR thermometry as a noninvasive monitoring technique that can provide real-time temperature measurements. MRgFUS was proven to minimize undesirable heating of the adjoining muscle and skin close to the target tumor and to increase penetration depths of the thermal reach [61,111]. Table 5 presents some studies that used different heating modes to trigger drug release from TSLs.

## 5. Concluding Remarks

Applying mild hypothermia (40–43 °C) to solid tumors, using different methods, results in the destruction of the structure of the cancer cells and affects other cellular processes, making those cells more prone to cancer treatments such as radiation and antineoplastic drugs. Mild hypothermia increases the blood flow, as well as the permeability of tumor vasculature. Such effects will enhance the extravasation of the NPs and homogenize their distribution within the deep tissues of the tumors. NPs designed to be thermosensitive will not only benefit from the advantages of applying mild hypothermia mentioned above, but will also employ this heat to trigger the release of their loaded drugs in a controlled manner. Drug delivery systems incorporating temperature-sensitive nanocarriers are promising therapeutic platforms with immense horizons for applications in different areas of medicine. Such nanocarrier-based systems can combat the shortcomings of traditional treatment methods. They incorporate nanoparticles that exhibit special biological properties and features that synergistically aid in the localized delivery and release of therapeutic agents at specific sites. Thermo-responsive systems have proven to be versatile, flexible, and tunable. Oncological hyperthermia is particularly interesting because research has proven synergistic interactions between temperature fluctuations and tumor survival pathways, providing grounds for potentially promising thermosensitive-based curative systems. Given the broadness and vastness of this field, this review’s scope is limited to thermosensitive liposomal systems, due to their relevance in clinical applications.

Among all nanocarriers, liposomes are the most established and have already made the transition from bench to bedside. Liposomes are highly stable, biocompatible, and biodegradable, with a unique ability to encapsulate both hydrophilic and hydrophobic drugs. Developing new types of liposomes that are thermosensitive exploits the inherent merits of liposomal systems, with the added benefits of thermal activation, to trigger the release of their loads. Liposomes are sensitive to temperature by nature, due to the structure of the hydrocarbon chains forming the phospholipid bilayer surrounding the liposomes. These unique characteristics of the phospholipids allow a reversible transformation between the gel phase and the liquid phase, depending on the surrounding temperature. This is known as the transition temperature (Tc), which depends on the length and saturation of the phospholipid chain. This is the basis of the traditional thermosensitive liposomes, or TTSL. The simple structure of the TTSL and the possibility of being directly triggered to release their load by hyperthermia paved the road for their progression. However, clinical trials showed that the effective release of Doxorubicin from TTSL requires a high temperature (above 42 °C), which is associated with some unwanted side effects, together with a slow release and lack of burst release kinetics. The new generation of thermosensitive liposomes includes the incorporation of thermosensitive phospholipids containing one acyl chain (LTSL) to stabilize the grain boundaries, leading to a quick and well-controlled drug release upon heating, or the incorporation of thermosensitive polymers (PTSL), which, upon heating, change their structure leading to disruption of the liposomal membrane and resulting in a controlled burst release of their loaded drugs.

Despite the promising in vivo results, progress to the medical application of thermosensitive NPs is still impeded, due to the many obstacles associated with the great complexity and heterogeneity of human tumors, compared to those of experimental animals. Achieving total drug release from thermosensitive liposomes, when exposed to heat, is a challenging task that needs to be optimized. Liposomes are allowed to extravasate through the leaky tumor vasculature (EPR effect) before accumulating inside the tumor. Mild hyperthermia is then applied to trigger drug release. It is essential that the heat is distributed equally within the heterogeneous tumor tissues, where the thermosensitive liposomes are scattered, while focusing the heating process only on the tumor, with no adverse effects on the neighboring tissues. Second, the heterogeneity of human tumors means that not all of the thermosensitive liposomes will be able to benefit from the EPR effect, and some will only pass through the blood vessels. It is important that those thermosensitive liposomes are also heated sufficiently as they quickly pass through the vessels surrounding the heated tumor. This will allow them to release their encapsulated drugs, resulting in damaging the blood vessels, which will enhance the therapeutic efficiency of the drug. Generally, it is important to control (i) the timing of the heating process, to ensure drug release from both the liposomes present inside the tumor and those passing through the tumor blood vessels; and (ii) the degree and duration of the applied heat, to ensure successful drug release, to a therapeutic level, while limiting the hyperthermia side effects and the development of drug resistance. Ensuring a successful treatment regime using thermosensitive liposomes and mild hyperthermia depends on optimizing all the different parameters to achieve successful clinical trials.

## Figures and Tables

**Table 1:** Volume change % and transition temperatures (in D_2_O or PBS) of some thermo-responsive Pluronic F127-based particles.

Material	Preparation Method	Volume Change (%)	Transition Onset (T °C)	Ref.
Pluronic^®^ F127/heparin	Modified emulsification/solvent evaporation method	~99	~25	[45]
Pluronic^®^ F127/poly(ethylenimine)	Modified emulsification/solvent evaporation method	92–97	~21	[43]
Au/Pluronic^®^ F127	Self-assembly then conjugation	~96	~18	[44]
Pluronic^®^ F127/PEG	Modified emulsification/solvent evaporation method	~89	~23	[42]

**Table 2:** Some traditional thermosensitive liposomal systems (TTSLs) and their corresponding transition temperatures.

Encapsulated Drug	Composition Molar Ratio	Experimental/Release Conditions	Findings	Ref.
Doxorubicin and MRI contrast agent [Gd(HPDO3A)(H_2_O)]	DPPC/HSPC/CH/DPPE-PEG_2000_/DOTA-DSPE 50:25:15:3:1	Hyperthermia was induced by exposing TSLs samples homogeneously distributed in a gel, by heating from 37 °C to 42 °C inside a clinical (magnetic resonance high intensity focused ultrasound) MR-HIFU setup.	Simultaneous and quantitative release of the drug and the MRI contrast agent was observed from the TSLs at 42 °C, while none was observed 37 °C after exposure for 1 h.	[61]
Doxorubicin	DPPC/DSPC/DPPGOG 50:20:30	TSLs were added to preheated fetal calm serum (FCS) or HN buffer where the temperature was varied from 37 to 45 °C over a time period of up to 180 min, and doxorubicin release was measured using fluorescence spectrometry.	89.1 ± 4.0% of doxorubicin over was retained in the TSLs for 3 h at 37 °C in the presence of serum. The release rate was significantly increased by incorporating DPPGOG.	[62]
Mitomycin C (MMC)	DPPC/DPPG 7:3	Diluted TSL samples were incubated in 30% (volume/volume) rat plasma for 60 min at the desired temperature (37 °C and 44 °C), then the released MMC was removed with cation exchange resin and the concentration of the retained drug in the liposomes was measured by high-performance liquid chromatography (HPLC).	The temperature-dependent content release efficiency % increased to 96% at the higher temperature. MMC leakage from the TSLs was suppressed in the presence of rat plasma and reached a plateau of 15%.	[63]
Pyrimidine Analogue Gemcitabine (dFdC)	DPPC/DSPC/DPPG_2_ 50:20:30	Diluted TSL samples were incubated in a preheated thermoshaker in FCS or HN for 5 min at 43 °C. After incubation, the samples were centrifuged where the filtrate containing the released drug was analyzed by HPLC.	The TSLs were stable at 37 °C in serum after 6 h of incubation and showed less than 20% release, while at 43 °C, 81.8 ± 15.0% of dFdC was released.	[64]
Vinorelbine	DPPC/MPPC/DSPE-PEG2000 86:5:4	In vitro WST-1 proliferation assay was used to evaluate the TSL dose-dependent effect and temperature on the viability of H22 cells. Cells were incubated for 72 h with the treatment at 37 °C and 42 °C.	Cells incubated at the higher temperature exhibited less cell viability%.	[65]
Doxorubicin and Vincristine	DPPC/DSPE-PEG_2000_/MSPC 75:17:8	Drug release from the TSLs was determined at 37 °C, 39 °C, 41 °C, and 42 °C over a period of 60 min. The time-dependent drug release profiles at 37 and 42 °C were assessed by HPLC.	Released amount of both payloads was about 85% within the first 5 min of heating at 42 °C from the TSLs, while less than 10% of the total drugs amount was released at 37 °C after heating for 30 min.	[66]
Docetaxel	DPPC/DSPE/PEG2000/EPC/MSPC 82:11:4:3:4	The TSLs were suspended in phosphate-buffered saline (PBS) 37 °C and 42 °C, then an analysis made done by dialysis.	In vitro drug release showed less drug released at 37 °C than at 42 °C, as after 2 h of incubation the TSLs released 15% and 40% of their load, respectively.	[67]
5-Fluorouracil (5-FU)	DPPC/CHO/DSPE-PEG 90:5:5	TSLs were diluted in PBS and exposed to temperatures varying from 25 to 49 °C over a time period of 30 min, in a heated water bath to determine time-dependent release. Temperature-dependent release was measured at each temperature in the range, by heating the samples for 10 min in Eppendorf tubes in heated water bath.	Drug release approached 70% as temperature increased from 37 °C to 49 °C.	[68]

Note: (DPPC) 1,2-Dipalmitoyl-sn-glycero-3-phosphocholine; (DSPC) 1,2-Distearoyl-sn-glycero-3-phosphocholine; (DSPE) 1,2-Distearoyl-sn-glycero-3-phosphoethanolamine; (DPPGOG) 1,2-dipalmitoyl-sn-glycero-3-phosphoglyceroglycerol (DPPG_2_) 1,2-Dipalmitoyl-sn-glycero-3-phospho-rac-glycerol; (MSPC) 1-Myristoyl-2-stearoyl-sn-glycero-3-phosphocholine.

**Table 4:** Summary of studies on the effect of copolymerization on LCST and liposomes Tc using the polymer PNIPAAm.

Comonomer	Liposome Composition	Encapsulated Drug	Modulation to Thermo-Responsiveness	Ref.
Free radical copolymerization of PNIPAAm with ODA	EPC	Calcein/carboxyfluorescein	The copolymer containing 1 mol% ODA had a LCST of 27 °C, compared to 32 °C for pure NIPAAm. ODA chains served as fixation sites of NIPAAm onto the core of the liposomes. Liposomes incorporating the copolymer exhibited enhanced thermosensitivity and showed more sustainable release profiles.	[92]
Free radical copolymerization of PNIPAAm with AAm	DOPE/EPC (6:4, *w*/*w*)	Calcein	Incorporating 10%, 20%, and 30% of AAm with NIPAAm increased the LCST to 39, 47, and 53 °C, respectively. Tuning the polymer LCST directly affects the liposomes T_c_. At T > 50 °C, the liposomal formulation incorporating 10% AAm showed 80% drug release.	[93]
Free radical copolymerization of PNIPAAm with AAm	DPPC	Doxorubicin	Increasing the AAm% in the copolymer from 17 to 24% resulted in increasing the LCST from 40 to 47 °C. The respective modified liposomal formulations exhibited a T_c_ similar to the copolymers’ LCST.	[91]
Free radical copolymerization with 3 structurally different comonomers: Apr, DMAM, NIPMAM	EPC	Calcein	The three copolymers’ LCST was ~40 °C. The transition enthalpy (ΔH) of the copolymers: NIPMAM > DMAM > Apr. Drug release % from the different modified liposomes increased as ΔH increased. The copolymer containing NIPMAM formed the highest hydrophobic domains above the LCST, which resulted in stronger interactions between the copolymer and the lipid bilayer; thus, augmenting perturbation upon heating, which caused the highest release.	[94]
Reversible-deactivation radical copolymerization with PAA	DPPC	Doxorubicin	Incorporating 5% PAA increased the copolymer LCST to 42 °C as its hydrophobicity increased. The modified liposomes were stable at physiological conditions, but released 70% and 100% after 5 min of heating at 40 °C and 42 °C, respectively.	[86]

Note: (PNIPAAm) Poly-N-isopropylacrylamide; (ODA) octadecyl acrylate; (AAm) acrylamide; (EPC) egg phosphocholine; (DOPE) dioleoylphosphatidylethanolamine; (Apr) N-acryloylpyrrolidine; (DMAM) N,N-dimethylacrylamide; (NIPMAM) N-isopropylmethacrylamide; (PAA) propyl acrylic acid.

**Table 5:** Summary of various studies using different heating modalities to trigger release from TSLs.

Liposomal Formulation	Heating Modality	Encapsulated Drug/Targeted Cancer	In Vivo Model	Response	Ref.
DPPC/DSPC/DSPE–PEG_2000_/70:25:5	Water bath	Doxorubicin/breast cancer	Orthotopic mice bearing breast tumors (MDA-MB-231 and T-47D)	The potency of neoadjuvant hyperthermia with TSLs was demonstrated, where heated tumors showed increased vascularization and permeability	[112]
DPPC/DSPC/DPPG_2_/50:20:30	Laser	Doxorubicin/soft tissue sarcoma	Brown Norway rats bearing syngeneic soft tissue sarcomas (BN175)	Heated tumors treated with TSLs showed more selective Doxorubicin uptake and accumulation	[113]
DPPC/DSPC/DPPG_2_/50:20:30	Laser	Doxorubicin/soft tissue sarcoma	Brown Norway rats bearing syngeneic soft tissue sarcomas (BN175)	Hyperthermal-triggered drug release from TSLs resulted in a 13-fold increase in Doxorubicin accumulation inside tumors.	[104]
DPPC/DSPC/DPPG_2_/50:20:30	HIFUS	Gemcitabine/soft tissue sarcoma	Brown Norway rats bearing syngeneic soft tissue sarcomas (BN175)	Combining HIFUS and TSLs showed distinguished tumor growth suppression	[64]
DPPC/MSPC/DSPE–PEG2000/DSPG/83:3:10:4	Water bath	Paclitaxel/lung cancer	Male Kunming mice bearing Lewis lung carcinoma (LLC)	Tumors treated with TSLs and exposed to heating experienced an arrest in growth	[67]
lyso-lecithin containing LTSLs	MR-HIFUS	Doxorubicin/squamous cell carcinoma	Rabbits bearing Vx2 carcinoma	LTSLs combined with MR-HIFUS enhanced tumor specificity and increased Dox uptake.	[114]

## Data Availability

No new data were created or analyzed in this study. Data sharing is not applicable to this article.
